# CAPN5 attenuates cigarette smoke extract-induced apoptosis and inflammation in BEAS-2B cells

**DOI:** 10.18332/tid/186183

**Published:** 2024-04-22

**Authors:** Herui Li, Yiming Ma, Tiao Li, Zihang Zeng, Lijuan Luo, Xiangming Liu, Yi Li, Yan Chen

**Affiliations:** 1 Department of Respiratory Medicine, The Second Xiangya Hospital, Central South University, Changsha, China; 2 Research Unit of Respiratory Diseases, Central South University, Changsha, China; 3 Clinical Medical Research Center for Respiratory and Critical Care Medicine in Hunan Province, Changsha, China; 4 Diagnosis and Treatment Center of Respiratory Disease, Central South University, Changsha, China; 5 Department of Infectious Disease, The Second Xiangya Hospital, Central South University, Changsha, China

**Keywords:** CAPN5, cigarette smoke, apoptosis, inflammation

## Abstract

**INTRODUCTION:**

Apoptosis and chronic inflammation are the main phenotypes in chronic obstructive pulmonary disease (COPD) pathogenesis. Cigarette smoke exposure is the leading risk factor for COPD, which causes aberrant airway epithelial structure and function. As a non-classical calpain, the molecular function of calpain5 (CAPN5) in COPD remains unclear. This study investigated the role of CAPN5 in mediating cigarette smoke extract (CSE)-induced apoptosis and inflammation.

**METHODS:**

Immunohistochemistry (IHC) and Western blotting (WB) were performed to detect the location and expression of CAPN5. In vitro, BEAS-2B cells were transfected with CAPN5 siRNA or CAPN5 plasmid, followed by phosphate-buffered saline (PBS) or cigarette smoke extract (CSE) treatment. The protein expression levels of CAPN5, NF-κB p65, p-p65, IκBα, p-IκBα and apoptosis proteins (BCL-2, BAX) were measured by WB. Flow cytometry (FCM) was performed to analyze the cell apoptosis index.

**RESULTS:**

CAPN5 was mainly expressed in the airway epithelium and significantly decreased in the COPD-smoker and emphysema-mouse groups. Silencing CAPN5 significantly decreased the protein expression of BCL-2, IκBα, and increased p-p65 and BAX protein expression. Additionally, an increased apoptosis index was detected after silencing CAPN5. Moreover, overexpression of CAPN5 partly inhibited IκBα degradation and p65 activation, and reduced CSE-induced inflammation and apoptosis.

**CONCLUSIONS:**

These combined results indicate that CAPN5 could protect against CSE-induced apoptosis and inflammation, which may provide a potential therapeutic target for smoking-related COPD.

## INTRODUCTION

Chronic obstructive pulmonary disease (COPD), one of the most common chronic respiratory diseases, is characterized by persistent respiratory symptoms and airflow limitation, and has high morbidity and mortality worldwide^1^. The pathogenesis of COPD is complicated, and current pathophysiological theories have determined that COPD derives from a set of different dynamic gene-environment interactions, which can occur throughout an individual’s life and modulate the development, maintenance, and function of the lungs^2,3^. Cigarette smoke (CS), a highly complex mixture that contains more than 7000 compounds and at least 70 carcinogens, is the critical environmental factor of COPD^4^. CS can cause continuous recruitment of immune cells, mucus secretion, and dysfunction of ciliary movement. Moreover, CS is the main source of exogenous oxides. It leads to a series of pathophysiological processes, including direct injury to lung tissue, protease-antiprotease imbalance, and activation of transcription factors to promote the inflammation, damage, and apoptosis of structural cells^5^. Discovering associated signaling or mediating factors might provide a useful strategy for alleviating lung inflammatory reactions and epithelial cell injury.

Calpains (CAPNs) are a class of calcium-dependent cysteine proteases encoded by 15 members: classical calpains (CAPN1–3, 8–9, and 11–14) and non-classical calpains (CAPN5–7, 10, and 15–16)^6^. In COPD pathogenesis, calpains are activated by CS, contributing to airway and pulmonary vascular remodeling^7,8^; however, these studies mainly focus on CAPN1, CAPN2, or the calpain-inhibitor calpeptin and have not reached a consensus^9^. CAPN5, one of the non-classical members of the calpain family, lacks the penta-EF-hand motif of classical calpains but retains catalytic and Ca2+-binding domains and contains a unique C-terminal domain^10^. CAPN5 is ubiquitously expressed and localizes to the cytoplasm and nucleus, and has been reported to participate in the development of neovascular vitreoretinopathy^11^, endometriosis^12^, and polycystic ovary syndrome^13^. However, the specific molecular functions and effects of CAPN5 in pulmonary and smoking-related diseases are unknown.

The redox-sensitive nuclear factor-kappaB (NF-κB) family of transcription factor proteins has a variety of transcriptional regulatory effects and plays critical roles in a wide array of biological processes, including cell proliferation, differentiation, apoptosis, stress response, immune cell activation, and oncogenesis^14^. Inhibitor of κB (iκB), which prevents NF-κB translocation to the nucleus and binding to DNA by masking its nuclear-localization sequence, regulates the activation of NF-κB^15^. High levels of activated NF-κB and decreased IκBα are observed in COPD patients compared with non-smoking healthy individuals^16^. Calpains can bind proline (P), glutamate (E), serine (S), and threonine (T), dubbed PEST domains, and promote degradation of IκBα by the calmodulin-like domain of the large subunit^17^; indeed, calpeptin attenuates CS-induced pulmonary inflammation by suppressing calpain/IκBα signaling in mice and BEAS-2B cells^18^. Given that classical calpains affect the development of COPD, we hypothesize that nonclassical calpain CAPN5 may play a crucial role in airway inflammation and apoptosis.

In this study, we determined the expression of CAPN5 *in vivo* and *in vitro*, and further detected downstream factors after silencing or overexpressing CAPN5. Thus, this study may illuminate how CAPN5 regulates CSE-induced inflammation and apoptosis in human bronchial epithelial cells.

## METHODS

### Subjects

Human lung tissue samples were acquired from patients who underwent lung resection as a consequence of carcinoma and were at least 5 cm away from the cancerous tissue. We included 17 subjects and divided them into three groups: nonsmokers without COPD as non-smokers (n=7), smokers without COPD as smokers (n=6), and smokers with COPD as COPD-smokers (n=4). Clinical information of the patients, such as age, sex, smoking history, and lung function, was collected and is summarized in Table 1. The diagnosis of COPD was followed by the Global Initiative for Chronic Obstructive Pulmonary Disease (GOLD) (http://www.goldcopd.com). Eligible criteria were previously reported in the study by Chen et al.^19^. The study was approved by the Research Medical Ethics Committee of the Second Xiangya Hospital of Central South University (Changsha, China). In accordance with the Declaration of Helsinki, written informed consent was obtained from all human subjects before enrollment.

**Table 1:** Baseline characteristics of participants

	*Non-smoker (N=7) Mean ± SD*	*Smoker (N=6) Mean ± SD*	*COPD-smoker (N=4) Mean ± SD*
Age (years)	53.29 ± 9.36	57.33 ± 15.77	57.75 ± 5.62
Male, n (%)	4 (57.1)	5 (83.3)	3 (75.0)
Smoking index (pack-years)	-	26.67 ± 14.72	32.50 ± 20.62
FEV1	2.91 ± 1.05	2.56 ± 0.37	1.78 ± 0.77*
FEV1/pred, %	102.46 ± 11.11	93.85 ± 12.42	61.38 ± 12.80*^†^
FEV1/FVC, %	84.96 ± 10.02	80.95 ± 6.74	57.99 ± 11.17*^†^

* p<0.05 compared with non-smokers.

† p<0.05 compared with smokers. COPD: chronic obstructive pulmonary disease. FEV1: forced expiratory volume in 1 s. FVC: forced vital capacity.

### CSE preparation

CSE was prepared in accordance with a previously described protocol by Chen et al.^19^ with minor modifications. Briefly, nonfiltered cigarettes (Tar: 11 mg, nicotine: 0.8 mg, Marlboro, Longyan Tobacco Industrial Co., Ltd., Fujian, China) were burned and the smoke passed through the solvent via a connection to a vacuum pump [i.e. the smoke of 1 cigarette through 10 mL of Dulbecco’s minimum essential medium (DMEM) for cell experiments or five cigarettes through 10 mL of phosphate-buffered saline (PBS) for animal experiments]. The filtered solution was considered 100% CSE and was serially diluted with culture medium or intraperitoneal injection for subsequent study. The CSE solution was prepared freshly for all experiments.

### Animals

Twenty-four six-week-old male specific-pathogenfree BALB/C mice (Slyke Jingda, Hunan, China) weighing 21–23 g were randomly divided into two groups (control group and emphysema group). The emphysema mouse model was established by intraperitoneal injection of 0.3 mL of CSE on Days 0, 11, and 22, while the control group was injected with 0.3 mL of PBS intraperitoneally at the same time, in accordance with the protocol of previous studies with slight modifications^20^. All mice were killed on Day 28, and lung tissues were obtained for further experiments described below.

### Immunohistochemistry (IHC)

Lung tissue samples were fixed in 4% formaldehyde for at least 24 h, embedded in paraffin, and cut into 3.5 μm thick sections. After antigen retrieval in citrate buffer (pH 6.0) for 10 min in a microwave, 0.3% hydrogen peroxide was applied to the samples for 10 min, and the sections were incubated with anti-calpain5 (1:20, sc-271271, Santa Cruz Biotechnology) at 4°C overnight. Samples were then incubated with goat anti-rabbit IgG antibody conjugated with peroxidase (Proteintech) for 30 min at room temperature. Diaminobenzidine (DAB) was added, and hematoxylin was used for counterstaining. The CAPN5 expression score was the average percentage of positive cells in fields under ×400 magnification calculated by five random images per section.

### Lung tissue morphometry

The sections were stained with hematoxylin and eosin (H&E). Emphysema of lung tissue samples was assessed by morphometry, which was quantified by the mean linear intercept (MLI) and destructive index (DI), as previously described by Zhang et al.^21^ and He et al.^22^.

### Cell culture

BEAS-2B cells were cultured in DMEM (Gibco, Thermo Fisher Scientific, Waltham, MA, USA) supplemented with 10% fetal bovine serum, 100 U/ mL penicillin, and 100 μg/mL streptomycin (Gibco, Thermo Fisher Scientific, Waltham, MA, USA) in a 37°C culture chamber with 5% CO2.

### Small-interfering RNA (siRNA) preparation and transfection

BEAS-2B cells were passed to a 6-well plate for 24 h to 40–60% confluence, then 50 nM negative control siRNA or CAPN5 siRNA (RiboBio, Guangzhou, China) was transfected by Lipofectamine™ 3000 Transfection Reagent (Invitrogen, Thermo Fisher Scientific, Waltham, MA, USA) for 48 h (mRNA) or 72 h (protein) following the manufacturer’s instructions.

### CAPN5 plasmid preparation and transfection

A CAPN5 plasmid (1 μg/mL) was introduced into BEAS-2B cells for 6 h using Lipofectamine™ 3000 Transfection Reagent (Invitrogen, Thermo Fisher Scientific, Waltham, MA, USA).

### Western blotting

Total protein of lung tissue or BEAS-2B cells was extracted by RIPA lysis buffer (Beyotime) with protease inhibitor (CWBio) and phosphatase inhibitor (CWBio) and measured by a BCA Protein Assay Kit (Thermo Fisher Scientific, Waltham, MA, USA). The denatured protein was separated using polyacrylamide gel electrophoresis (CWBio) and transferred onto PVDF membranes (Millipore, USA). These membranes were incubated overnight with antibodies at 4°C: β-actin (1:2000, 20536-1-AP, Proteintech), anti-calpain5 (1:200, sc-271271, Santa Cruz Biotechnology), BCL-2 (1:1000, 3498, Cell Signaling Technology), BAX (1:1000, 505992, Proteintech), IκBα (1:1000, 4812, Cell Signaling Technology), p-IκBα (Ser32) (1:1000, 2859, Cell Signaling Technology), NF-κB p65 (1:1000, 4764, Cell Signaling Technology), and p-p65 (Ser536) (1:1000, 3033, Cell Signaling Technology), followed by incubation with HRP-labeled goat anti-rabbit or anti-mouse secondary antibody (1:5000, Proteintech). The bound complexes were visualized by the ECL plus Western blotting detection system (Bio-Rad, USA). Band densities were quantified using ImageJ software.

### Flow cytometry (FCM) analysis

BEAS-2B cells in 6-well plates were washed twice with cold PBS and resuspended in 100 μL of 1× binding buffer. Five microliters of APC Annexin V and 5 μL of 7-AAD were added to the cell suspension, gently vortexed, and incubated for 15 min at room temperature in the dark. Finally, 200 μL of 1× binding buffer was added to the cell suspensions and analyzed using flow cytometry within one hour.

### Statistical analysis

The categorical variables are presented as count (percentage) and were analyzed using the chi-squared test or Fisher’s exact test. The continuous variables are presented as the mean ± standard deviation (SD) and were analyzed by Student’s t-test or one-way analysis of variance (ANOVA) combined with Tukey’s *post hoc* test using GraphPad Prism 8 software (San Diego, CA, USA). Significance was determined at p<0.05.

## RESULTS

### CAPN5 expression is decreased in lung tissues of COPD-smokers

The baseline characteristics of the eligible subjects are presented in Table 1. No significant difference was found in age or sex among the three groups. In contrast to non-smokers, the FEV1 was significantly lower in COPD-smokers. The values of FEV1pred and FEV1/FVC in COPD-smokers were significantly decreased in comparison with those in non-smokers and smokers.

To clarify the location and expression of CAPN5, we examined CAPN5 protein in human lung tissue specimens. In lung tissues of non-smokers, IHC showed that CAPN5 was expressed in the airway epithelium and localized within the nucleus of epithelial cells (Supplementary file Figure 1a). Compared with non-smokers, the protein abundance of CAPN5 was significantly lower (p<0.05) in the lung tissues of smokers and COPD-smokers (Supplementary file Figure 1b). The protein level of CAPN5 was significantly decreased in the lung tissue of COPD smokers compared to that of non-smokers (Supplementary file Figures 1c and 1d).

**Figure 1:**
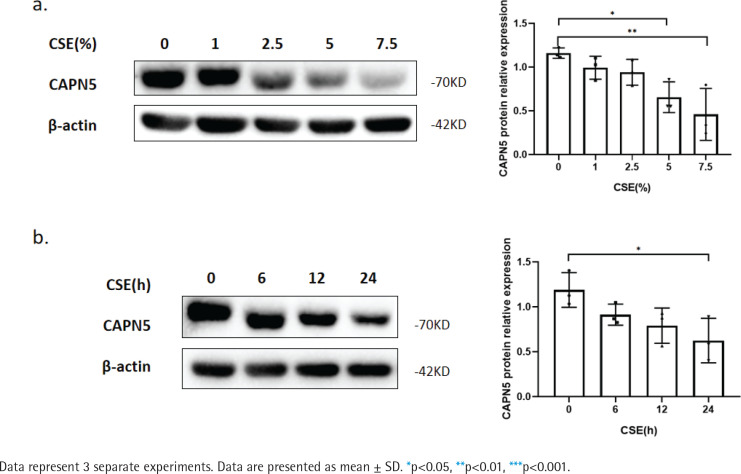
CSE reduces CAPN5 protein expression in BEAS-2B cells: a) BEAS-2B cells were exposed to 0, 1, 2.5, 5, and 7.5% CSE; Cell lysates were analyzed for CAPN5 or β-actin expression by immunoblotting; b) BEAS-2B cells were treated with 5% CSE for 0, 6, 12, and 24 h; Cell lysates were for CAPN5 or β-actin expression by immunoblotting

### CSE diminishes CAPN5 protein expression in emphysema mice

To demonstrate the effect of CSE on CAPN5 *in vivo*, we established experimental emphysema mouse models by intraperitoneal injection of CSE^19^. H&E staining was conducted to analyze lung morphology. Destruction of the alveolar wall and loss of the alveolar unit were observed in mice intraperitoneally injected with CSE, suggesting that emphysema models were well built (Supplementary file Figure 2a). The morphologically quantitative analysis of the sections showed significantly higher MLI (Supplementary file Figure 2b) and DI (Supplementary file Figure 2c) in the emphysema group compared with the control group. In lung specimens of emphysema mice, IHC showed that CAPN5 was located in the bronchial epithelium, and its expression was markedly decreased in the emphysema group compared with the control group (Supplementary file Figures 2d and 2e). These results were confirmed using WB, which demonstrated a similar decrease in CAPN5 protein (Supplementary file Figures 2f and 2g).

**Figure 2:**
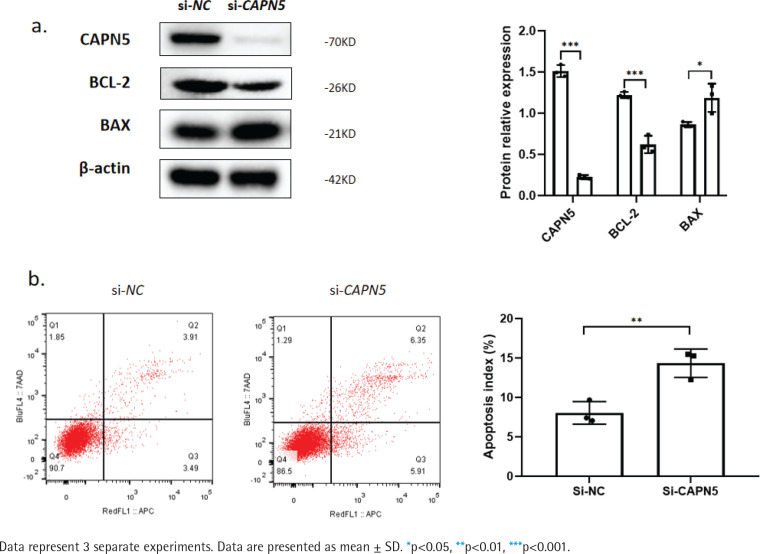
Silencing CAPN5 promotes the apoptosis of BEAS-2B cells. Negative control (NC) and CAPN5 small-interfering RNA (siRNA) were delivered into BEAS-2B cells using Lipofectamine 3000 reagent for 72 h: a) Cell lysates of the si-NC and si-CAPN5 groups were analyzed for CAPN5, BCL-2, BAX, or β-actin expression by immunoblotting; b) Apoptosis analysis of BEAS-2B cells in the si-NC and si-CAPN5 groups using Annexin V-APC/7-AAD staining and FCM

**Figure 3:**
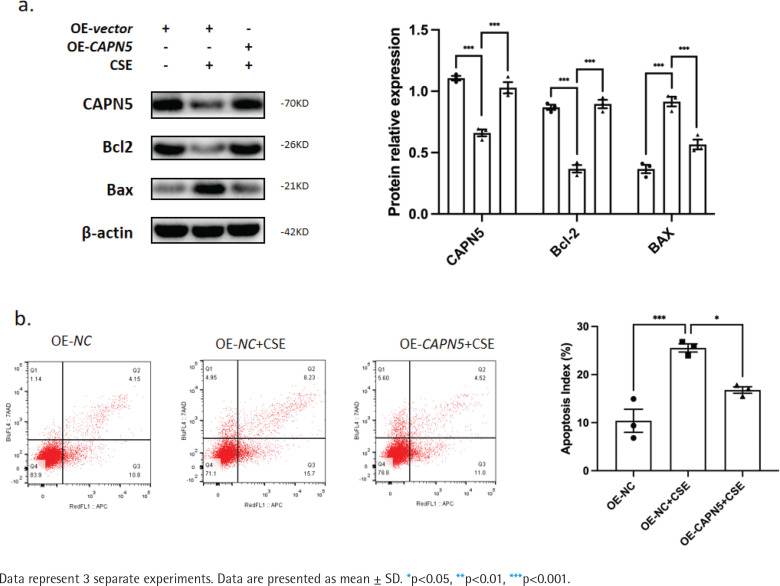
Overexpressing CAPN5 prevents CSE-induced apoptosis of BEAS-2B cells. Vector- or CAPN5-overexpressing plasmids were separately transfected into BEAS-2B cells. Before the cells were collected, the transfected cells were stimulated with PBS or 5% CSE for 24 h: a) Cell lysates of the oe-vector, oe-vector+CSE and oe-CAPN5+CSE groups were analyzed for CAPN5, BCL-2, BAX, or β-actin expression by immunoblotting. The densitometry results of CAPN5, BCL-2, and BAX protein expression; b) Apoptosis analysis of BEAS-2B cells in oe-vector, oe-vector+CSE and oe-CAPN5+CSE groups using Annexin V-APC/7-AAD staining and FCM

### CSE reduces CAPN5 protein expression in BEAS2B cells

To further verify whether CSE decreases the CAPN5 level *in vitro*, we primarily conducted CSE treatment in human bronchial epithelial BEAS-2B cells^19^. The WB results showed that CSE reduced the protein levels of CAPN5 in both concentration- and timedependent manners (Figures 1a and 1b).

### Silencing CAPN5 promotes the apoptosis of BEAS-2B cells

Cigarette smoke induces lung epithelial cell dysfunction and death, contributing to the pathogenesis of COPD^19^. To investigate whether decreased CAPN5 affected apoptosis and inflammation of BEAS-2B cells, we conducted loss-of-function assays using siRNA that exhibited significant reductions in CAPN5 mRNA and protein levels. CAPN5 knockdown significantly decreased anti-apoptotic protein (BCL-2) and increased pro-apoptotic proteins (BAX) (Figure 2a). FCM analysis showed that the apoptosis index of BEAS-2B cells was significantly increased in the siCAPN5 group compared with the si-NC group (Figure 2b).

### CAPN5 prevents CSE-induced apoptosis in BEAS-2B cells

To further clarify whether CAPN5 could prevent CSEinduced apoptosis, a CAPN5 overexpressing plasmid was transfected into BEAS-2B cells, followed by PBS or 5% CSE treatment for 24 h. The results from WB and FCM analyses indicated that CSE decreased the expression of an anti-apoptotic protein (BCL-2) and increased the expression levels of pro-apoptotic proteins (BAX) (Figure 3a), as well as the apoptosis index of BEAS-2B cells (Figure 3b). Conversely, overexpressing CAPN5 partially attenuated CSE-induced apoptosis in BEAS-2B cells.

### CAPN5 mediates the activation of inflammation via the NF-κB/IκBα signaling pathway in BEAS-2B cells

**Figure 4:**
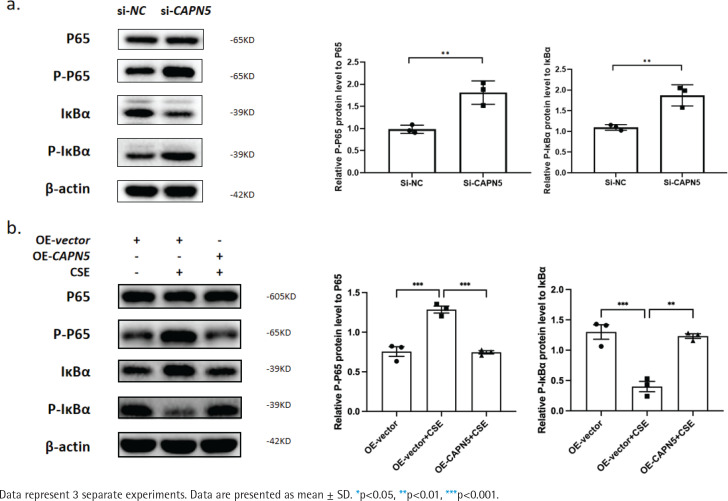
CAPN5 mediates the activation of inflammation via the NF-κB/IκBα signaling pathway in BEAS-2B cells: a) Cell lysates of the si-NC and si-CAPN5 groups were analyzed for CAPN5, p65, p-p65, IκBα, p-IκBα or β-actin expression by immunoblotting; b) Cell lysates of the oe-vector, oe-vector+CSE and oe-CAPN5+CSE groups were analyzed for CAPN5, p65, p-p65, IκBα, p-IκBα or β-actin expression by immunoblotting

Cigarette smoke is known to cause oxidative stress in the airway epithelium^23^, resulting in the activation of transcription factors and the release of inflammatory mediators, following damage and dysfunction of the airway barrier, contributing to the pathogenesis of COPD^24^. To explore the possible mechanisms by which CAPN5 regulates CSE-induced inflammation, we hypothesized that NF-κB/IκBα may be a potential signaling pathway by which CAPN5 regulates CSE-induced apoptosis and inflammation. As shown in Figures 4a and 4b, we found that knocking down CAPN5 and 5% CSE treatment significantly increased p-p65 protein expression and promoted IκBα phosphorylation, and overexpression of CAPN5 significantly decreased the ratios of p-p65 to p65 and p-IκBα to IκBα. Overall, these results suggested that CAPN5 impaired IκBα phosphorylation and NF-κB activation in CSE-induced apoptosis and inflammation.

## DISCUSSION

Our results demonstrate that CAPN5 is significantly decreased in lung samples of COPD patients and emphysema mice, as well as in CSE-treated BEAS-2B cells. *In vitro*, silencing CAPN5 promoted apoptosis and inflammation in BEAS-2B cells, and overexpression of CAPN5 attenuated CSE-induced inflammation and apoptosis. Furthermore, phosphorylation of IκBα and activation of NF-κB may be potential mechanisms of this process. Given the above, CAPN5 protects against CSE-induced apoptosis and inflammation by suppressing IκBα phosphorylation and NF-κB activation, which could be potential COPD therapy targets.

Calpains, a family of 15 Ca^2+^-activated neutral proteases widely located in the cytosol and mitochondria of ubiquitous tissues, are involved in a broad range of cellular functions and human diseases. Calpian5 is encoded by CAPN5, which is ubiquitously expressed and implicated in the pathogenesis of a wide range of human diseases; however, its specific function remains enigmatic. CAPN5 mutations are associated with autoimmune uveitis, retinal neovascularization, and photoreceptor degeneration^11^. Sáez et al.^25^ determined that CAPN5 might increase risks for cardiovascular diseases (such as diastolic blood pressure and cholesterol levels). In human endometrial cells, calpain5 expression is downregulated by decreasing HOXA10, which promotes the development of endometriosis through apoptosis inhibition^12^.

We showed decreased CAPN5 expression levels in airway epithelial cells after CSE treatment. Heijink et al.^26^ found that CSE induces calpain-dependent disruption of intercellular contacts via epidermal growth factor receptor (EGFR) activation. Tobacco also induces phosphorylation of both μ- and m-calpain in association with their activation, increased migration, and invasion of lung cancer cells^27^. In contrast, incubation of pulmonary artery endothelial cells with CSE results in a dose-dependent decrease in calpain activity and causes inhibition of tube formation, migration, proliferation, and endothelial monolayer wound repair^9^. Fei et al.^28^ reported that oxidizing conditions may inhibit calpain activity and further affect a series of biological processes. Cigarette smoke consists of various oxidative gases, which are the main sources of exogenous oxides and lead to oxidant/antioxidant imbalance and oxidative stress. Thus, CAPN5 was downregulated *in vivo* and *in vitro* in our study, indicating that CSE may reduce the expression of CAPN5 in human bronchial epithelial cells via oxidative modification.

Calpain is an abundant cytoplasmic protease that can cleave many intracellular signaling and structural proteins and has a number of different roles in cells^29,30^. Most current reports describe that active calpains induce apoptosis by mediating BH3-interacting domain death agonist (Bid) cleavage, cytochrome c release, apoptosome formation, and caspase three activation^31^. Conversely, CAPN3 deficiency induces myonuclear apoptosis^32^, and overexpressing μ-calpain reduces the activity of caspase-3 and apoptosis in chronic lymphocytic leukemia (B-CLL) cells^33^. In a recent study by Łopatniuk et al.^34^, calpain activity in B-CLL cells was associated with decreased activities of pro-apoptotic caspases-3 and -9 and reciprocally with increased anti-apoptotic BCL-2. In our research, CAPN5 silencing decreased the expression of BCL-2, increased the expression of BAX and cleaved caspase-3, and promoted the apoptosis of BEAS-2B cells while overexpressing CAPN5 correspondingly reduced cell apoptosis. Thus, we concluded that CAPN5 may protect BEAS-2B cells from apoptosis by degrading proapoptotic factors.

Our results show that CSE-induced CAPN5 deficiency promotes IκBα degeneration, NF-κB activation, and inflammatory cytokine release, while overexpression of CAPN5 protects against inflammation. Apoptosis and inflammation are a series of pathophysiological processes that fuel each other during the pathogenesis of COPD. Indeed, Kim et al.^35^ reported that active calpain induced downregulation of the transcription factors NF-κB, STAT3, and STAT5. Interestingly, calpains have been mostly reported to be involved in inflammation in a variety of human diseases, and inhibition of calpains reduces the inflammatory response through the NF-κB signaling pathway^36^, but the interaction between calpain5 and the NF-κB signaling pathway has not been well described. Considering the synergistic or antagonistic functions of each member of the calpain family, we propose that CAPN5 may stimulate phosphorylated degradation of IκBα and activation of NF-κB, thus suppressing CSE-induced inflammation and apoptosis. Relevant experiments to intervene CAPN5 in COPD animal models are needed to investigate the relationship between CAPN5 and COPD further.

## CONCLUSIONS

We observed significant decreases in CAPN5 in COPD-smokers, emphysema mice, and CSE-treated BEAS-2B cells. Overexpression of CAPN5 reduces apoptosis and inflammation of BEAS-2B cells by inhibiting IκBα degeneration and NF-κB phosphorylation. This is the first study to investigate the role of CAPN5 in CSE-induced inflammation and apoptosis, which reveals CAPN5 as a potential gene target involved in the pathogenesis of COPD.

## Supplementary Material



## Data Availability

The data supporting this research are available from the authors on reasonable request.
