# Supplementary material for: CAPN5 attenuates cigarette smoke extract-induced apoptosis and inflammation in BEAS-2B cells
Source: Tob Induc Dis. 2024 Apr 22;22:10.18332/tid/186183. doi: 10.18332/tid/186183 (PMC11033979; doi:10.18332/tid/186183)
Supplement: Supplementary file 1 [file TID-22-65-s1.pdf]

Supplementary Figure 1. CAPN5 is decreased in the lung tissues of COPD- smokers. a. Detection of CAPN5 in peripheral lung tissues of subjects. CAPN5-positive cells were frequently distributed in the airway epithelium of nonsmokers but were rarely distributed in which of the smokers and COPD- smokers; The mean IOD of CAPN5 in the airway epithelium. b. Lung tissues of the subjects were lysed for CAPN5 and β-actin immunoblotting. Results are shown with a representative of n= 3 experiments. Data are presented as the mean ± SD. *$P < 0.05$; ** $P < 0.01$; *** $P < 0.001$.

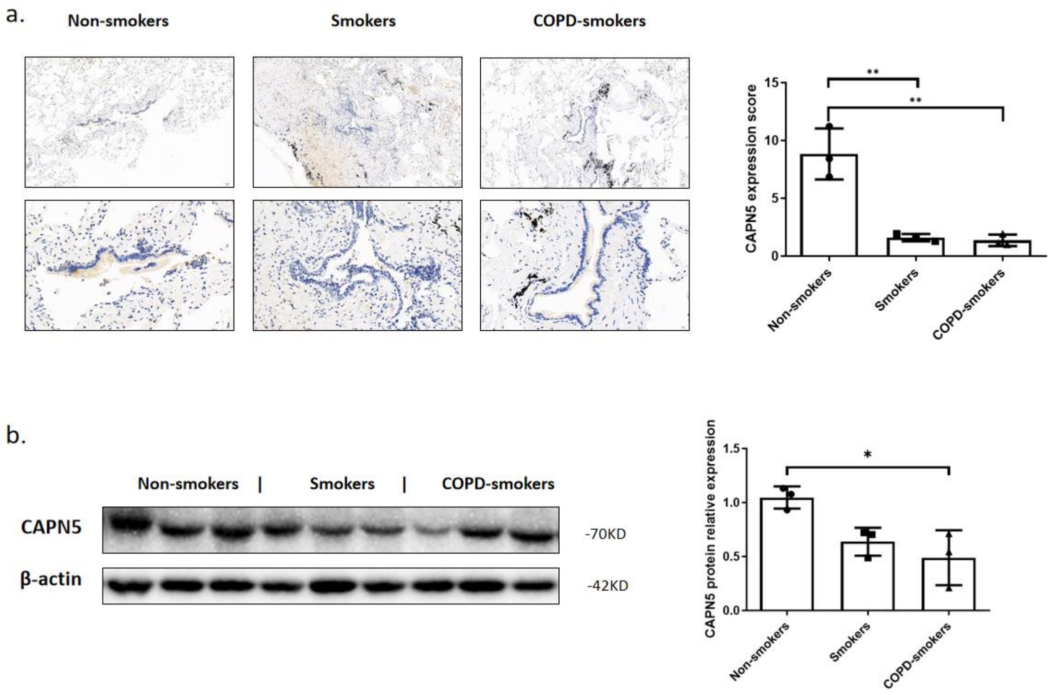

Supplementary Figure 2. CSE diminishes CAPN5 protein expression in emphysema mice.

a. Photomicrographs of lung tissues of mice in the control and emphysema groups stained with hematoxylin and eosin. Morphometric measurements of b. the mean linear intercept (MLI, μm) and c. the destructive index (DI, %) in the two groups. d. Representative staining of CAPN5 in lung tissues of mice with immunohistochemical (IHC) analysis. CAPN5-positive cells were more frequently distributed in the airway epithelium of the control group than in the emphysema group; The mean IOD of CAPN5 in the mouse lung tissues. e. Lung tissues of the subjects were lysed for CAPN5 and β-actin immunoblotting. Results are shown with a representative of more than 3 times experiments. Data are presented as the mean ± SD. *$P < 0.05$; ** $P < 0.01$; *** $P < 0.001$.

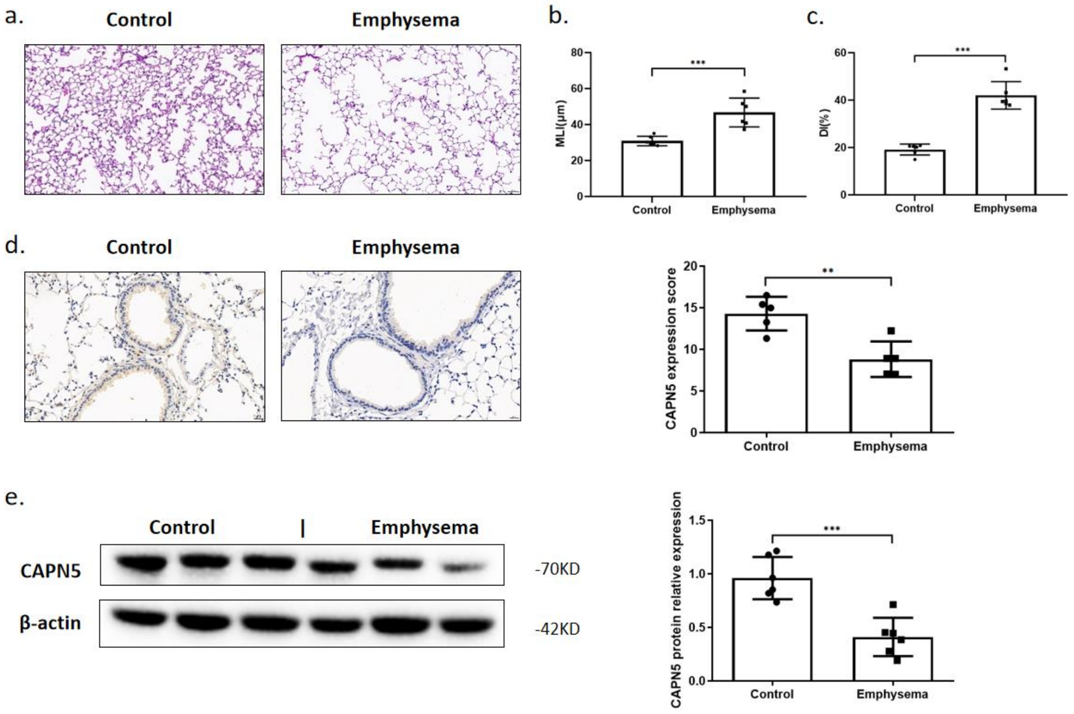

© 2024 Li H. et al.
